# FS‐Mamba: Feature‐wise scanning Mamba UNet for automatic image segmentation in liver tumor radiotherapy

**DOI:** 10.1002/acm2.70677

**Published:** 2026-07-07

**Authors:** Peijun Yin, Xueren Zhang, Xin Liu, Zekun Jiang, Qingtao Qiu, Yong Yin, Zhenjiang Li

**Affiliations:** ^1^ Department of Radiation Physics Shandong Cancer Hospital and Institute, Shandong First Medical University and Shandong Academy of Medical Sciences Jinan China; ^2^ School of Science Sun Yat‐sen University Shenzhen China; ^3^ Department of Gynecological Radiotherapy Harbin Medical University Cancer Hospital Harbin China; ^4^ West China Biomedical Big Data Center West China Hospital, Sichuan University Chengdu China

**Keywords:** deep learning, frequency domain features, liver cancer radiotherapy, Mamba, medical image segmentation, online adaptive radiotherapy, state space model

## Abstract

**Background:**

Medical image segmentation is fundamental to radiotherapy planning, yet accurate delineation of organs at risk and tumor targets remain challenging due to anatomical variability and low soft‐tissue contrast in CT images.

**Purpose:**

To develop a lightweight, high‐precision automatic segmentation network that meets the dual clinical requirements of accuracy and computational efficiency for online adaptive liver cancer radiotherapy.

**Methods:**

We propose FS‐Mamba, a U‐shaped encoder^21^‐decoder network built upon a novel Frequency‐Long SSM Block that integrates three innovations: (1) a flip‐selective scanning strategy (FS‐Scanning) that dynamically reorders feature sequences to prioritize salient information; (2) a frequency‐domain modeling branch using Fast Fourier Transform to enhance boundary discrimination; and (3) a long‐memory state space model (LSSM) that augments the A‐matrix to strengthen historical state retention. The model was validated on three datasets: the public CT‐ORG (140 CT volumes, 6 organs) and Synapse (30 cases, 8 organs) benchmarks, and an in‐house liver cancer dataset (50 patients, 12 structures including PTV and GTV). Comparisons were made against UNet, nnUNet, TransUNet, Swin‐UMamba, and the clinically mainstream TotalSegmentator using Dice similarity coefficient (DSC), 95th percentile Hausdorff distance (HD95), and subjective physicist scoring.

**Results:**

FS‐Mamba achieved mean DSC/HD95 of 92.81%/10.74 on CT‐ORG, 81.28%/14.32 on Synapse^30^, and 92.68%/10.17 on the liver cancer dataset, outperforming all compared methods with statistical significance (*p* < 0.05). It received the highest mean subjective clinical score of 4.69/5.0 from 10 blinded medical physicists, approaching the ground‐truth reference (4.94/5.0). Despite its leading accuracy, FS‐Mamba maintains the smallest model size (20.57 M parameters, 31.94 GFLOPs) and fastest inference (20.16 ms per case), representing a 4.5x parameter reduction and 6.5x speedup compared to TotalSegmentator.

**Conclusions:**

FS‐Mamba provides a clinically viable solution for automated multi‐organ and tumor target segmentation that satisfies the stringent time constraints of online adaptive radiotherapy while maintaining expert‐level accuracy.

## 1 INTRODUCTION

Medical image segmentation plays a foundational role in medical image analysis by dividing an image into multiple segments, each representing meaningful anatomical structures or areas of interest.^1^, ^2^ In radiotherapy, organ segmentation is one of the core technologies for achieving precise radiotherapy.^3^, ^4^ It provides fundamental support for the design, optimization, and evaluation of treatment plans by accurately delineating key anatomical structures (such as tumors and organs at risk) in medical images. Firstly, organ segmentation helps clinicians precisely locate the tumor target area and reduces mis‐irradiation of surrounding normal tissues. For example, in head‐and‐neck or abdominal radiotherapy, segmenting the boundaries between tumors and adjacent organs (such as the spinal cord and kidneys) is crucial for formulating dose distributions.^4^, ^5^, ^6^, ^7^ Secondly, by segmenting organs at risk (such as the heart, lungs, and liver), radiotherapy planning systems (such as Eclipse and RayStation) can automatically calculate dose‐limiting conditions, thereby minimizing the risk of radiation‐induced damage while ensuring the tumor receives an appropriate dose.^8^, ^9^, ^10^, ^11^ Additionally, organ segmentation supports the formulation of personalized radiotherapy strategies. Based on the dynamic changes in patient‐specific anatomical structures (such as respiratory motion and tumor regression), adaptive radiotherapy requires real‐time or inter‐fraction organ segmentation results to dynamically adjust the irradiation plan.^8^, ^12^, ^13^, ^14^ However, accurate segmentation of medical images remains challenging due to the significant variability in the shape, size, location, and texture of different human tissues and lesions.^15^, ^16^ Additionally, some tissues or lesions exhibit fuzzy boundaries and low contrast, making them difficult to distinguish accurately.^4^ Therefore, further advancements are essential to enhance segmentation accuracy, which is crucial for reliable diagnosis, treatment planning, and monitoring in clinical practice.

Currently, deep learning‐based methods dominate the field of medical image segmentation, which can be broadly categorized into CNN‐based and Transformer‐based approaches. Expanding the model's receptive field is a critical factor in enhancing segmentation performance. CNN‐based methods struggle to model global relationships due to the locality of the convolution layer.^17^ In contrast, the transformer architecture,^18^, ^19^ which utilizes a self‐attention module to extract global information, has been extensively explored for medical image segmentation. TransUNet was the first to leverage the feature learning capability of the Vision Transformer (ViT)^20^ within a UNet encoder.^21^ Swin‐UNet and DCSUnet further explored the U‐shaped structure, utilizing Swin Vision Transformer network blocks exclusively.^22^, ^23^ While ViT performs well in capturing long‐range dependencies, the high computational overhead from self‐attention is not conducive to processing high‐resolution biomedical images.^24^, ^25^

To address the challenges of long sequence modeling, Mamba,^26^ derived from state space models (SSMs), is specifically designed to capture long‐range dependencies while improving the efficiency of training and inference. Compared to Transformers, these models scale linearly or near‐linearly with sequence length, all while preserving the ability to model long‐range dependencies, thereby achieving state‐of‐the‐art performance in continuous long‐sequence data analysis.^26^ In the context of computer vision, the applicability of Mamba is realized through VMamba, which leverages a Visual State Space (VSS) Block that combines the Cross Scanning Module (CSM) with the Selective State Space Model (S6).^27^

The CSM in VMamba converts two‐dimensional features into interleaved one‐dimensional features by scanning the feature map four times vertically and horizontally, enabling the Mamba structure to be applied to 2D images. However, due to the inherent limitations of this scanning method in capturing both long‐ and short‐term dependencies, the Mamba model experiences imbalances in interpreting image features, leading to suboptimal performance in detailed image segmentation. To address this challenge, various scanning methods, such as left‐skewed and right‐skewed scanning, depth scanning, zigzag scanning, and global‐plus‐local scanning, have been proposed.^28^ While these novel scanning techniques have significantly improved performance, they also result in increased computational overhead, which has become a notable drawback.

In this paper, we propose Feature‐wise Scanning Mamba UNet (FS‐Mamba), which integrates a flip‐selective scanning strategy (FS‐Scanning) based on image features, local frequency domain feature extraction and the long memory expansion technique from state space models (SSMs). The FS‐Scanning dynamically reorders features, allowing the SSM to focus on significant image characteristics rather than short‐term memory effects. The long memory expansion technique ensures robust inference capabilities, even with large images. Furthermore, by combining local frequency domain feature extraction with the SSM's ability to capture long‐range dependencies, FS‐Mamba achieves a better grasp of global features, reduces parameter count, and enhances segmentation boundary identification.

## 2 METHODS

### 2.1 Architecture overview

The proposed FS‐Mamba architecture utilizes a multi‐scale U‐Net structure with FSM Blocks as the core components. It begins with a Patch Embedding layer that splits the image into patches, similar to ViT and VMamba, while resizing the features to an arbitrary dimension, denoted as c ^20^, ^27^ The encoder performs multi‐layer feature extraction through multiple FSM Blocks. The decoder, organized in a U‐shaped structure, also consists of FSM Blocks, enabling it to combine features of the same size from the encoder. This design compensates for the spatial details lost during downsampling. Finally, a linear projection layer restores the resolution necessary for the final segmentation results.

### 2.2 FS‐scanning

The Mamba model's long‐term memory capability primarily relies on its State Space Model (SSM) to capture global information. However, as SSM sequentially aggregates previous information while processing features, the details of earlier information are gradually compressed as the model handles longer sequences. This irreversible compression and loss of detailed information directly leads to degraded accuracy and robustness in capturing long‐range dependencies, which in turn undermines the model's performance on downstream tasks such as fine‐grained medical image segmentation.

**FIGURE 1:**
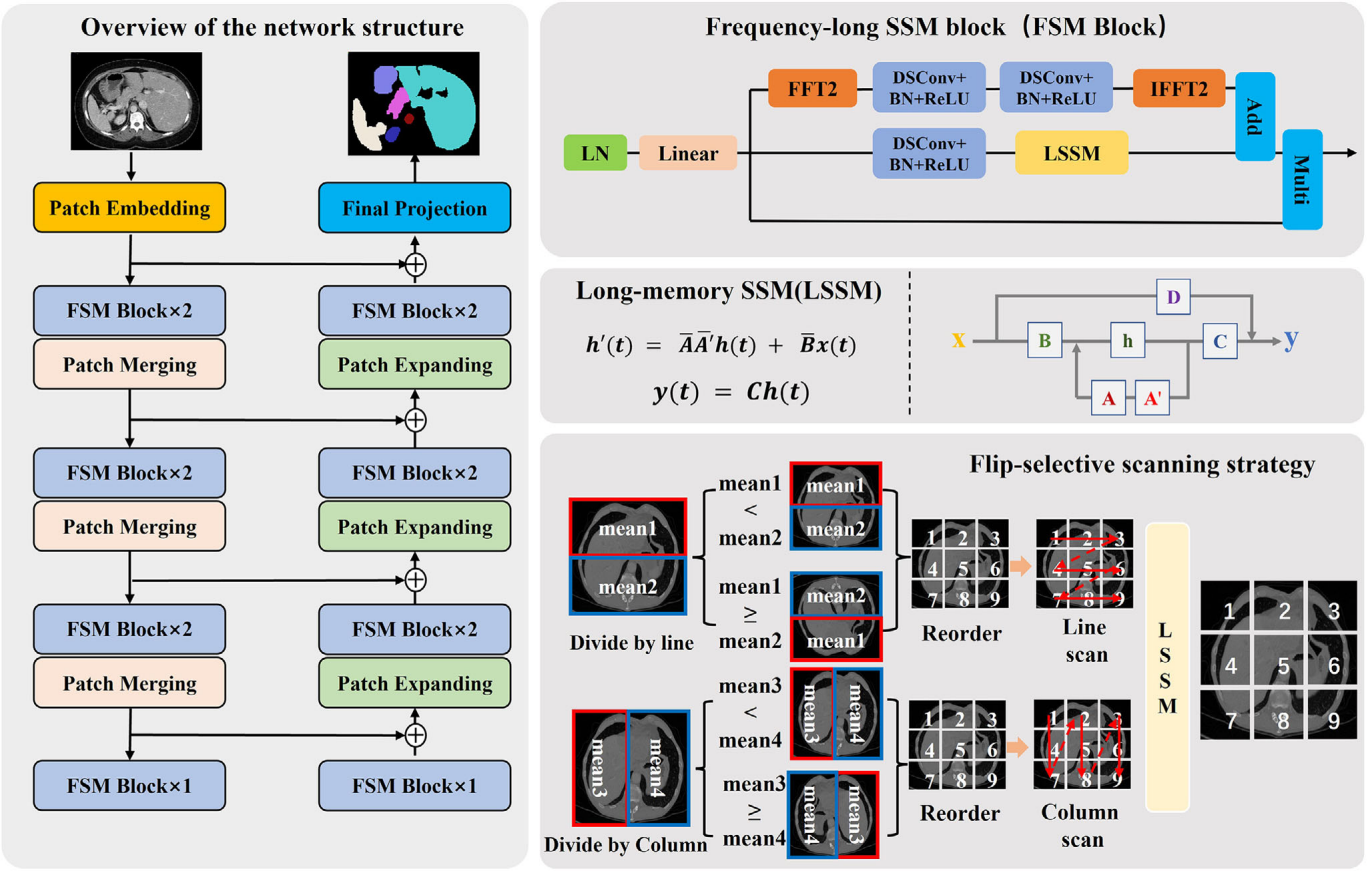
FS‐Mamba follows a U‐shaped architecture and consists of an encoder, a decoder, and skip connections. The encoder and decoder are constructed from FSM Blocks, which utilize frequency domain features to assist LSSM feature extraction. The LSSM employs the A’ matrix for A‐matrix augmentation. Additionally, FS‐Scanning flips based on the feature mean, placing emphasis on the end of the sequence to minimize compression loss.

Since the feature maps undergo nonnegative normalization via the BN layer and the ReLU activation function prior to LSSM, and the positive integer values of the labels are constrained to remain positive in conjunction with the Dice loss, the segmentation targets tend to have higher feature values. As shown in Figure 1, we reduce the scans in VMamba from four to two, using only one forward line scan and one forward column scan. Taking the line scan as an example, we first divide the feature map into two parts based on the number of lines, calculate the mean value of the features in each part, and then, by comparing these mean values, position the part with the larger mean at the end of the sequence before inputting it into the LSSM. Similarly, for the column scan, we calculate the mean value based on the number of columns and position the part with the larger mean at the end of the sequence. Assuming that W, H are the number of rows and columns of the feature map $F_{\mathrm{in}}$, i, i are the row indexes and column indexes respectively, the total number of pixels is n, $F_{\mathrm{inw}}$ is the $F_{\mathrm{in}}$ after row reset, $F_{\mathrm{inh}}$ is the $F_{\mathrm{in}}$ after column reset, $\mathrm{Fli}p_{w}$ and $\mathrm{Fli}p_{h}$ are the flip rows and flip columns, and $F_{\mathrm{out}}$ is the final output, V represents the pixel value of the point, the process can be described by the equation as follows:

(1) $$F_{\mathrm{inh}}=\left\{\begin{array}{cc} F_{\mathrm{in}}, & \sum_{i=1}^{\frac{W}{2}-1}\sum_{j=1}^{H}V_{ij}<\sum_{i=\frac{W}{2}}^{W}\sum_{j=1}^{H}V_{ij}\\ \mathrm{Fli}p_{h}\left(F_{\mathrm{in}}\right), & \sum_{i=1}^{\frac{W}{2}-1}\sum_{j=1}^{H}V_{ij}>\sum_{i=\frac{W}{2}}^{W}\sum_{j=1}^{H}V_{ij} \end{array}\right.\;,$$

(2) $$F_{\mathrm{inw}}=\left\{\begin{array}{cc} F_{\mathrm{in}}, & \sum_{i=1}^{W}\sum_{j=1}^{\frac{H}{2}-1}V_{ij}<\sum_{i=1}^{W}\sum_{j=\frac{H}{2}}^{H}V_{ij}\\ \mathrm{Fli}p_{w}\left(F_{\mathrm{in}}\right), & \sum_{i=1}^{W}\sum_{j=1}^{\frac{H}{2}-1}V_{ij}>\sum_{i=1}^{W}\sum_{j=\frac{H}{2}}^{H}V_{ij} \end{array}\right.\;,$$

(3) $$F_{\mathrm{out}}=\mathrm{Merging}\left(\mathrm{LSSM}\left(F_{\mathrm{inw}}\right),\mathrm{LSSM}\left(F_{\mathrm{inh}}\right)\right),$$

### 2.3 Frequency‐long SSM block

As shown in Figure 1, the FSM Block, which integrates mixed spatial and frequency domain features, consists of three branches. The first branch uses Fast Fourier Transform and Inverse Transform to extract frequency domain features. These are then combined with the features extracted by the second branch, the LSSM, and multiplied with the original features from the third branch to produce the final output for auxiliary selective long memory state space model (LSSM) feature extraction. This module enhances the FSM Block's feature extraction capability by utilizing comprehensive frequency domain features, thereby helping the model better identify boundaries.

#### 2.3.1 Fourier modeling branch

Unlike the spatial domain, frequency domain features can highlight boundaries more clearly, thus helping the model to accurately capture intricately organized edges. For the Fourier Modeling Branch, the input feature $F_{\mathrm{in}}$ first transforms into its Fourier spectrum via the Fast Fourier Transform (FFT) that produce the amplitude spectrum $A(F_{\mathrm{in}})$. The amplitude spectrum undergoes refinement through a Depthwise Separable Convolution Block(DSConvBlock) composed of two Depthwise Separable Convolution(DSConv), ultimately returning to the spatial domain via inverse fast Fourier transform (iFFT):

(4) $$F_{P}=\mathrm{iFFT}\left(\mathrm{DSConvBlock}\left(A\left(F_{\mathrm{in}}\right)\right)\right),$$

#### 2.3.2 Long‐memory SSM (LSSM)

To adapt to large‐resolution segmented images, we enhance the selective state space model (S6) by strengthening the memorization ability of the A‐matrix, resulting in what we call the selective long memory state space model (LSSM). This enhancement provides the LSSM with a stronger historical state memory capability. After discretization, the SSM‐based model can be computed using either linear recursion or global convolution, defined as:

(5) $$h^{\prime }\;\left(t\right)=\bar{A}\;h\left(t\right)+\bar{B}x\left(t\right),$$

(6) $$y\;\left(t\right)=Ch\left(t\right),$$

(7) $$\bar{K}=\left(C\bar{A},C\bar{A}\bar{B},\text{…},C{\bar{A}}^{L-1}\bar{B}\right)\;,$$

(8) $$y=x*\bar{K},$$

(9) $$y_{k}=C{\bar{A}}^{k}\bar{B}x_{0}+C{\bar{A}}^{k-1}\bar{B}x_{1}+\cdots +C\bar{A}\bar{B}x_{k-1}+C\bar{B}x_{k},$$

(10) $$y_{3}=\left(\begin{array}{cccc} C{\bar{A}}^{3}\bar{B} & C{\bar{A}}^{2}\bar{B} & C\bar{A}\bar{B} & C\bar{B} \end{array}\right)\;\left(\begin{array}{c} x_{0}\\ x_{1}\\ x_{2}\\ x_{3} \end{array}\right),$$

where, x(t)∈R, reaches the output y(t)∈R through the intermediate implicit state $h\left(t\right)\in R^{N}$, $A\in R^{N\times N}$ denotes the state matrix, $B\in R^{N\times 1}$, $C\in R^{N\times 1}$ denotes the projection parameters, A, B are the discrete parameters that A and B are transformed into using the discretization rule. $K\in R^{L}$ denotes a structured convolutional kernel and L denotes the length of the input sequence x.

A is the condensed essence of all previous historical information (which can be represented by a matrix of coefficients), in order to update the spatial state h(t) at the next moment based on A. We use the transpose of matrix $A^{\prime }$ to augment the A matrix by multiplying the two matrices to construct a more variable combination of coefficients than by directly increasing the dimension of the A matrix. Then our LSSM model can be expressed as follows:

(11) $$h^{\prime }\;\left(t\right)=\bar{A}\;\bar{A^{\prime }}h\left(t\right)+\bar{B}x\left(t\right),$$

Then the output is computed through a global convolution, defined as:

(12) $$\bar{K}=\left(C\bar{A}\bar{A^{\prime }},C\bar{A}\bar{A^{\prime }}\bar{B},\text{…},C{\bar{A}}^{L-1}{\bar{A^{\prime }}}^{L-1}\bar{B}\right)\;,$$

(13) $$y=x*\bar{K},$$

The multiplication of the two parameter matrices allows the A matrix to be augmented while also accommodating various methods of feature compression. The complete flowchart of the algorithm is shown in Figure 1. The D‐matrix is a parameter matrix that connects the input directly to the output using residual connections.

Ultimately, the computation of the Frequency‐long SSM block can be expressed as:

(14) $$F_{\mathrm{FSM}}=\left[F_{p}+y\left(t\right)\right]\;\times l\left(t\right),$$

l(t), is the initial input to the block.

## 3 EXPERIMENTS AND RESULTS

### 3.1 Datasets

In this study, three datasets were used to validate the performance of the FS‐Mamba model, including the CT‐ORG^29^ and Synapse^30^ public datasets as well as the private liver cancer dataset collected by our institute. We describe the three datasets in detail below.

#### 3.1.1 CT‐ORG

The CT‐ORG dataset consists of 140 CT images across six organ categories: liver, lung, bladder, kidney, bone, and brain. Of the 140 image volumes, 131 were dedicated CT scans, and 9 were CT components collected during PET‐CT examinations. Each image was from a different patient. Most images showed benign or malignant liver lesions, while some depicted metastases from breast, colon, bone, and lung cancers. Manual soft tissue labeling was performed on all images using ITK‐SNAP and morphological segmentation. We used images from 119 patients as the training set and the remaining 21 as the test set.

#### 3.1.2 Synapse

Synapse is a publicly available multi‐organ segmentation dataset containing 3779 axial abdominal clinical CT images from 30 abdominal CT cases, covering eight abdominal organs (aorta, gallbladder, left kidney, right kidney, liver, pancreas, spleen, and stomach). Following the setup of previous works,^22^ 18 of these samples were randomly selected as the training set, while the remaining 12 samples were used as the test set.

#### 3.1.3 Liver cancer dataset

The liver cancer dataset is sourced from our own research center. Professional radiation oncologists meticulously outlined the organs at risk (OARs) and target volumes within this dataset. It comprises eleven segmentation categories, namely the spleen, pancreas, stomach, liver, right kidney, left kidney, duodenum, spinal cord, heart, planning target volume (PTV), and gross tumor volume (GTV). We incorporated data from 50 patients into our dataset. Specifically, data from 40 patients were allocated for the training phase, while data from the remaining 10 patients were reserved for testing. The images of each patient were captured at a resolution of 512 × 512 × 200.

### 3.2 Implementation details

All our experiments were performed on NVIDIA GeForce RTX 3090 GPUs. The resolution of the CT‐ORG dataset was 512 × 512, and the batch size during training was set to 6. The training was conducted for 400 epochs. We used the Adam optimizer with = 0.9 and = 0.999 were used. The initial learning rate is 0.01 and the decay is 0.00003. The resolution of Synapse^30^ dataset was 224 × 224. We set the batch size to 20 and used the AdamW optimizer with = 0.9 and = 0.999, The initial learning rate is 0.01 and the decay is 0.00001. The training epoch was set to 400. For the liver cancer dataset, the batch size was set to 6 and the learning rate was 0.0001. The resolution was 512 × 512. We used the Adam optimizer with = 0.9 and = 0.999 to train the model. All training uses the Dice loss function.

### 3.3 Comparison with state‐of‐the‐art methods

We compare FS‐Mamba in detail with several benchmark methods, including UNet,^17^ nnUNet,^31^ TransUNet,^21^ Swin‐UMamba^32^ and TotalSegmentator.^33^ To quantitatively assess segmentation accuracy across multiple anatomical regions, we systematically computed the Dice similarity coefficient (DSC) and the 95th percentile Hausdorff distance (HD95) for all organ categories on three distinct datasets: the publicly available CT‐ORG and Synapse^30^ benchmark datasets, as well as our institutional liver cancer CT dataset. These metrics provide a unified framework for evaluating inter‐organ segmentation consistency and cross‐dataset generalization performance. We performed quantitative evaluation of all methods on complete 3D cases.

**FIGURE 2:**
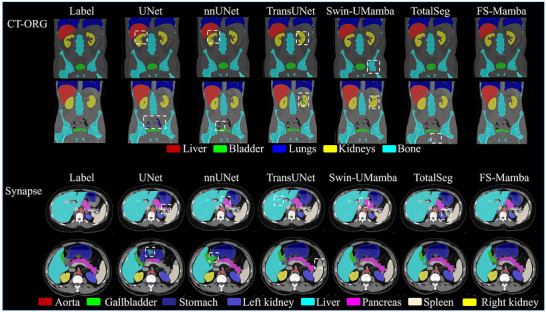
Comparison results of FS‐Mamba with other models on CT‐ORG and Synaspe datasets, with white boxes marking several regions that differ significantly from the reference image. FS‐Mamba has fewer segmentation errors on organs and tissues.

Figure 2 illustrates the segmentation performance of all models (including the clinically mainstream TotalSegmentator) on the CT‐ORG and Synapse^30^ datasets, with white boxes marking regions with significant deviations from the reference labels. On the CT‐ORG dataset, UNet, nnUNet, TransUNet, and Swin‐UMamba show varying intra‐organ pixel errors, blurred boundaries and scattered false‐positive fragments. TotalSegmentator delivers good contour integrity for high‐contrast large organs (liver and lungs), but presents notable deviations in fine boundaries of small organs and low‐contrast tissues. By contrast, FS‐Mamba achieves minimal intra‐organ segmentation errors, effectively suppresses false‐positive fragments, and enables accurate boundary delineation of large organs via the long‐term memory capability of LSSM. For the complex abdominal multi‐organ segmentation task on the Synapse dataset, the comparative models show frequent soft tissue misclassification and discontinuous segmentation regions. TotalSegmentator maintains stable overall segmentation for large organs with clear boundaries, but fails to accurately depict fine edges of low‐contrast organs (e.g., gallbladder and pancreas) and soft tissue junctions. FS‐Mamba significantly reduces cross‐organ soft tissue misclassification, and its frequency‐domain feature fusion ensures more continuous segmentation regions without extra noise compared with Swin‐UMamba, thus achieving superior segmentation quality.

The quantitative assessment results are shown in Tables 1 and 2, with segmentation performance presented as mean ± standard deviation, and statistical significance evaluated via two‐tailed paired Wilcoxon signed‐rank test at the patient level. The *p*‐values were classified as follows: ^*^ *p* < 0.05, ^**^ *p* < 0.01, ^***^ *p* < 0.001. On the CT‐ORG dataset, TransUNet achieved favorable liver and lung segmentation performance. TotalSegmentator, as the state‐of‐the‐art multi‐organ segmentation baseline, ranked second overall, demonstrating competitive performance across most organs and achieving an average Dice of 92.26% ± 1.49% and mean HD95 of 11.62% ± 2.95%, outperforming Swin–UMamba (Dice 91.64% ± 1.61%, HD95 12.05 ± 3.29), TransUNet (Dice 91.39% ± 1.78%, HD95 11.74 ± 2.56), nnUNet^31^ (Dice 91.06% ± 1.95%, HD95 11.82 ± 3.65) and UNet (Dice 86.98% ± 2.34%, HD95 13.94 ± 3.92). Our proposed FS‐Mamba outperformed all comparative methods in bladder, kidney and bone segmentation, and yielded the highest average Dice similarity coefficient (92.81% ± 1.27%) and lowest mean HD95 distance (10.74% ± 2.73%) across the 5 categories, with statistically significant improvements over all comparative models (all *p* < 0.05), including the strong baseline TotalSegmentator and most notably versus UNet.

**TABLE 1:** Quantitative evaluation of models in terms of Dice on the CT‐ORG and Synaspe dataset (mean ± standard deviation).

Model	Dice↑ (CT‐ORG Dataset)					
	Liver	Bladder	Lungs	Kidneys	Bone	Mean
UNet	93.34 ± 1.87^0.002^ ^**^	75.81 ± 3.52^0.000^ ^***^	92.12 ± 1.26^0.003^ ^**^	88.89 ± 2.19^0.001^ ^**^	84.74 ± 2.87^0.000^ ^***^	86.98 ± 2.34^0.001^ ^**^
nnUNet	95.57 ± 0.94^0.018^ ^*^	86.13 ± 3.17^0.003^ ^**^	94.88 ± 0.68^0.003^ ^**^	92.03 ± 1.62^0.004^ ^**^	86.67 ± 2.21^0.002^ ^**^	91.06 ± 1.95^0.003^ ^**^
TransUNet	**95.61 ± 1.23** ^0.037^ ^*^	85.47 ± 2.91^0.006^ ^**^	**95.97 ± 0.89** ^0.029^ ^*^	92.11 ± 1.47^0.004^ ^**^	87.81 ± 2.45^0.002^ ^**^	91.39 ± 1.78^0.002^ ^**^
Swin‐UMamba	93.73 ± 1.65^0.009^ ^**^	85.91 ± 2.48^0.005^ ^**^	95.92 ± 0.71^0.032^ ^*^	93.39 ± 1.33^0.002^ ^**^	89.25 ± 2.09^0.002^ ^**^	91.64 ± 1.61^0.002^ ^**^
TotalSeg	93.12 ± 1.07^0.042^ ^*^	86.18 ± 2.76^0.027^ ^*^	95.72 ± 0.95^0.035^ ^*^	94.12 ± 1.18^0.019^ ^*^	92.15 ± 1.82^0.012^ ^*^	92.26 ± 1.49^0.015^ ^*^
FS‐Mamba	92.49 ± 0.88	**86.35 ± 2.24**	95.51 ± 0.73	**94.75 ± 0.93**	**94.96 ± 1.41**	**92.81 ± 1.27**

Dice↑ (Synapse Dataset)						
	Aorta	Gallbladder	Kidney (L)	Kidney (R)	Liver	Pancreas
UNet	89.07 ± 2.15^0.002^ ^**^	69.72 ± 4.38^0.000^ ^***^	77.77 ± 3.76^0.001^ ^**^	68.60 ± 4.21^0.000^ ^***^	93.43 ± 1.42^0.003^ ^**^	53.98 ± 5.17^0.000^ ^***^
nnUNet	87.37 ± 1.69^0.002^ ^**^	67.41 ± 4.05^0.002^ ^**^	83.69 ± 3.24^0.003^ ^**^	79.63 ± 3.58^0.003^ ^**^	92.16 ± 1.57^0.002^ ^**^	55.31 ± 4.82^0.001^ ^**^
TransUNet	85.47 ± 2.37^0.005^ ^**^	66.53 ± 4.29^0.003^ ^**^	83.28 ± 3.45^0.004^ ^**^	79.61 ± 3.72^0.003^ ^**^	**94.29 ± 1.18** ^0.041^ ^*^	56.58 ± 4.63^0.003^ ^**^
Swin‐UMamba	84.98 ± 2.51^0.005^ ^**^	68.52 ± 3.94^0.003^ ^**^	85.35 ± 3.12^0.027^ ^*^	**81.27 ± 3.41** ^0.039^ ^*^	93.53 ± 1.36^0.002^ ^**^	57.09 ± 4.75^0.002^ ^**^
TotalSeg	87.56 ± 1.93^0.023^ ^*^	69.38 ± 4.16^0.031^ ^*^	**84.89 ± 3.07** ^0.037^ ^*^	80.97 ± 3.35^0.029^ ^*^	93.91 ± 1.27^0.033^ ^*^	58.72 ± 4.58^0.017^ ^*^
FS‐Mamba	**90.02 ± 1.63**	**70.15 ± 3.47**	84.42 ± 2.57	80.67 ± 2.89	94.24 ± 0.98	**60.27 ± 4.05**

Spleen	Stomach	Mean				
86.67 ± 2.42^0.002^ ^**^	75.58 ± 3.89^0.000^ ^***^	76.85 ± 3.56^0.001^ ^**^				
87.49 ± 2.17^0.003^ ^**^	77.91 ± 3.64^0.002^ ^**^	78.87 ± 3.29^0.002^ ^**^				
**90.66 ± 1.84** ^0.035^ ^*^	76.60 ± 3.97^0.003^ ^**^	79.13 ± 3.15^0.002^ ^**^				
88.16 ± 2.28^0.002^ ^**^	76.95 ± 3.72^0.002^ ^**^	79.48 ± 3.38^0.002^ ^**^				
87.82 ± 2.05^0.025^ ^*^	80.24 ± 3.51^0.019^ ^*^	80.44 ± 3.21^0.021^ ^*^				
87.45 ± 1.78	**83.01 ± 3.04**	**81.28 ± 2.65**				

* *p* < 0.05

** *p* < 0.01

*** *p* < 0.001, versus FS‐Mamba (two‐tailed paired Wilcoxon signed‐rank test at the patient level). *P*‐value (DSC): Based on patient‐level average Dice across all organs.

**TABLE 2:** Quantitative evaluation of models in terms of HD95 on the CT‐ORG and Synapse^30^ dataset (mean ± standard deviation).

Model	HD95↓ (CT‐ORG Dataset)					
	Liver	Bladder	Lungs	Kidneys	Bone	Mean
UNet	12.21 ± 4.82^0.002^ ^**^	20.54 ± 9.76^0.000^ ^***^	14.37 ± 5.64^0.003^ ^**^	11.85 ± 4.61^0.001^ ^**^	10.72 ± 4.02^0.000^ ^***^	13.94 ± 3.92^0.001^ ^**^
nnUNet	10.73 ± 3.27^0.018^ ^*^	18.32 ± 6.89^0.003^ ^**^	9.89 ± 3.82^0.042^ ^*^	10.21 ± 3.12^0.004^ ^**^	9.97 ± 2.60^0.038^ ^*^	11.82 ± 3.65^0.003^ ^**^
TransUNet	10.21 ± 3.89^0.037^ ^*^	15.92 ± 3.31^0.045^ ^*^	11.92 ± 4.26^0.029^ ^*^	11.43 ± 3.72^0.004^ ^**^	9.23 ± 3.21^0.002^ ^**^	11.74 ± 2.56^0.002^ ^**^
Swin‐UMamba	10.81 ± 2.94^0.009^ ^**^	17.74 ± 5.97^0.005^ ^**^	11.65 ± 3.50^0.032^ ^*^	10.72 ± 2.81^0.002^ ^**^	9.31 ± 2.42^0.002^ ^**^	12.05 ± 3.29^0.002^ ^**^
TotalSeg	**9.90 ± 2.35** ^0.042^ ^*^	16.83 ± 4.52^0.027^ ^*^	10.47 ± 2.54^0.035^ ^*^	10.96 ± 2.25^0.031^ ^*^	9.94 ± 1.89^0.012^ ^*^	11.62 ± 2.95^0.015^ ^*^
FS‐Mamba	10.24 ± 1.56	**15.56 ± 8.23**	**9.15 ± 1.82**	**9.58 ± 1.49**	**9.19 ± 2.17**	**10.74 ± 2.73**

HD95↓ (Synapse Dataset)						
	Aorta	Gallbladder	Kidney (L)	Kidney (R)	Liver	Pancreas
UNet	15.21 ± 5.13^0.002^ ^**^	27.83 ± 12.45^0.000^ ^***^	19.62 ± 8.76^0.001^ ^**^	16.94 ± 7.23^0.000^ ^***^	15.32 ± 5.27^0.003^ ^**^	27.49 ± 13.89^0.000^ ^***^
nnUNet	13.73 ± 3.48^0.002^ ^**^	22.79 ± 8.26^0.002^ ^**^	16.27 ± 5.94^0.003^ ^**^	12.95 ± 4.89^0.003^ ^**^	12.13 ± 3.56^0.002^ ^**^	23.82 ± 10.23^0.001^ ^**^
TransUNet	13.56 ± 4.12^0.005^ ^**^	24.78 ± 10.56^0.003^ ^**^	17.53 ± 7.43^0.004^ ^**^	16.07 ± 5.87^0.003^ ^**^	**11.92 ± 4.21** ^0.041^ ^*^	25.34 ± 11.76^0.003^ ^**^
Swin‐UMamba	13.92 ± 1.86^0.035^ ^*^	21.47 ± 7.29^0.003^ ^**^	15.88 ± 5.32^0.027^ ^*^	**13.21 ± 3.98** ^0.039^ ^*^	13.25 ± 3.82^0.002^ ^**^	22.69 ± 9.34^0.002^ ^**^
TotalSeg	13.30 ± 2.31^0.023^ ^*^	22.19 ± 5.70^0.031^ ^*^	11.90 ± 3.94^0.037^ ^*^	13.40 ± 2.97^0.029^ ^*^	12.09 ± 2.13^0.033^ ^*^	18.75 ± 6.89^0.017^ ^*^
FS‐Mamba	**12.24 ± 1.67**	**17.81 ± 3.89**	**11.63 ± 2.15**	14.43 ± 2.68	13.94 ± 3.12	**17.92 ± 4.23**

Spleen	Stomach	Mean				
14.67 ± 6.34^0.002^ ^**^	17.67 ± 7.56^0.000^ ^***^	19.34 ± 5.37^0.001^ ^**^				
12.51 ± 4.29^0.003^ ^**^	14.64 ± 5.12^0.002^ ^**^	16.11 ± 4.64^0.002^ ^**^				
**11.85 ± 5.12** ^0.035^ ^*^	12.91 ± 6.23^0.003^ ^**^	16.75 ± 5.50^0.002^ ^**^				
12.19 ± 3.87^0.002^ ^**^	14.32 ± 4.97^0.002^ ^**^	15.87 ± 3.99^0.002^ ^**^				
12.53 ± 2.86^0.025^ ^*^	14.68 ± 3.95^0.019^ ^*^	14.85 ± 3.69^0.021^ ^*^				
12.53 ± 2.04	**14.03 ± 2.56**	**14.32 ± 2.40**				

* *p* < 0.05

** *p* < 0.01

*** *p* < 0.001, versus FS‐Mamba (two‐tailed paired Wilcoxon signed‐rank test at the patient level). *P*‐value (HD95): Based on patient‐level average HD95 distance across all organs.

For the Synapse dataset covering eight abdominal organ segmentation tasks, TotalSegmentator maintained its second‐place performance, achieving an overall 8‐class mean Dice of 80.44% ± 3.21% and mean HD95 of 14.85 ± 3.69, which was superior to all other comparative models except FS‐Mamba. Notably, TotalSegmentator demonstrated robust performance particularly in the pancreas and stomach, achieving the second‐highest Dice for the stomach (80.24% ± 3.51%) and competitive results for the pancreas (58.72% ± 4.58%). FS‐Mamba exhibited clear advantages: it achieved higher Dice coefficients than competing methods on the aorta, gallbladder, left kidney, pancreas and stomach, and obtained the highest overall 8‐class mean Dice (81.28% ± 2.65%) and lowest mean HD95 (14.32 ± 2.40), with statistically significant gains over all comparative models (all *p* < 0.05 for both DSC and HD95), including TotalSegmentator. All comparisons between FS‐Mamba and the other models reached statistical significance, with the majority exhibiting *p* < 0.01 or *p* < 0.001. These findings preliminarily validate the performance and application potential of our method in medical image segmentation tasks.

**FIGURE 3:**
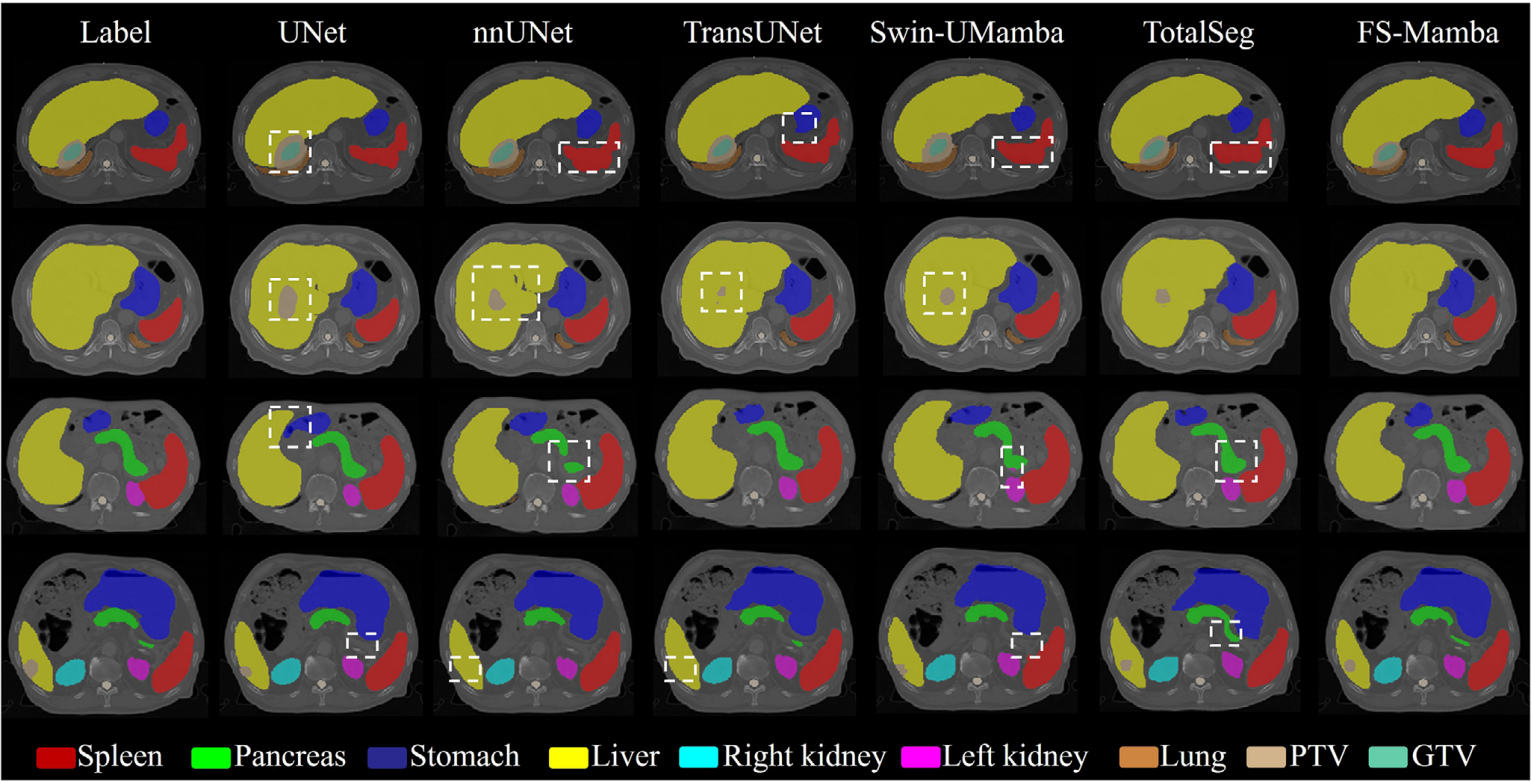
Comparison results of FS‐Mamba with other models on liver cancer dataset, with white boxes marking several regions that differ significantly from the reference image. The recognition accuracy of FS‐Mamba in both organs and tumor target areas is superior to that of other models.

As illustrated in Figure 3, a comparative assessment of FS‐Mamba against UNet, nnUNet,^31^ TransUNet, Swin‐UMamba, and the clinically mainstream TotalSegmentator on the liver cancer dataset highlights its distinct performance advantages. Visually, in the regions marked by white boxes, all comparative models exhibit noticeable segmentation deviations: UNet and nnUNet show obvious organ boundary misclassification and fragmented segmentation in tumor target areas, while TotalSegmentator, despite delivering relatively intact contouring for major organs, presents non‐negligible inaccuracies in the fine delineation of tumor target volumes (GTV/PTV) and the junction of adjacent low‐contrast tissues. In comparison, FS‐Mamba demonstrates superior recognition accuracy: inside the organ parenchyma, it minimizes pixel‐level errors, ensuring strong alignment with the reference labels; for tumor target regions and organ boundaries, it achieves precise delineation, effectively avoiding the extraneous noise or ambiguous boundary issues present in other models. Particularly, while TransUNet and Swin–UMamba show incomplete or misaligned segmentations in certain highlighted areas, FS‐Mamba maintains coherent and aggregated segmented regions at both organ and tumor target sites. This visual comparison underscores FS‐Mamba's excellence in handling the complex anatomical structures of the liver cancer dataset, outperforming all comparative models in terms of segmentation precision and structural integrity for both organs and tumor target volumes.

Tables 3 and 4 present the quantitative segmentation results on the real clinical liver cancer dataset, with all metrics reported as mean ± standard deviation. To mitigate the impact of inter‐organ anatomical heterogeneity, we comprehensively report the Dice coefficient, 95% Hausdorff Distance (HD95), and corresponding ‐values for each organ individually. Statistical significance was verified using the two‐tailed paired Wilcoxon signed‐rank test at the patient level, and all performance differences between the proposed FS‐Mamba and comparison models reached statistical significance (*p* < 0.05). Benefiting from the synergistic effect of frequency‐domain feature fusion and the long‐memory SSM block, our FS‐Mamba model achieves the best overall performance in both Dice and HD95 metrics. Specifically, FS‐Mamba yields the highest Dice values in 10 out of 12 organs, with a mean Dice of 92.68% ± 2.79%, and achieves the lowest average HD95 of 10.17 ± 1.29. The frequency‐domain branch significantly improves the boundary delineation for low‐contrast organs such as the pancreas (88.31% ± 3.46%, 12.81 ± 3.04) and duodenum (92.06% ± 3.49%, 10.92 ± 2.37). High‐frequency features captured by FFT enhance the segmentation precision of fine structures and reduce boundary errors, which also contributes to superior tumor target segmentation for GTV (85.29% ± 5.02%) and PTV (76.75% ± 6.03%). Meanwhile, the long‐memory SSM block strengthens the capability of long‐range dependency modeling, further boosting the segmentation accuracy and boundary stability of large organs including the liver (96.71% ± 1.69%, 9.43 ± 2.33) and stomach (95.47% ± 2.40%, 6.63 ± 1.52).

**TABLE 3:** Quantitative comparison of segmentation models in terms of Dice on the liver cancer dataset.

Model	Dice↑ (Liver Cancer Dataset)					
	Spleen	Pancreas	Stomach	Liver	Right Kidney	Left Kidney
UNet	94.88 ± 2.17^0.000^ ^***^	86.15 ± 4.09^0.000^ ^***^	91.00 ± 3.24^0.012^ ^**^	95.31 ± 2.03^0.004^ ^**^	96.04 ± 1.82^0.033^ ^*^	96.90 ± 1.66^0.003^ ^**^
nnUNet	95.94 ± 1.98^0.006^ ^**^	87.70 ± 3.88^0.028^ ^*^	92.57 ± 3.09^0.003^ ^**^	96.57 ± 1.82^0.029^ ^*^	96.93 ± 1.63^0.035^ ^*^	97.47 ± 1.49^0.009^ ^**^
TransUNet	96.00 ± 1.89^0.031^ ^*^	86.60 ± 3.92^0.004^ ^**^	93.14 ± 2.93^0.008^ ^**^	95.63 ± 1.93^0.037^ ^*^	96.89 ± 1.69^0.008^ ^**^	97.14 ± 1.58^0.039^ ^*^
Swin‐UMamba	95.89 ± 2.02^0.006^ ^**^	88.29 ± 3.72^0.042^ ^*^	93.37 ± 2.82^0.011^ ^**^	96.08 ± 1.88^0.044^ ^*^	96.82 ± 1.73^0.009^ ^**^	97.16 ± 1.55^0.045^ ^*^
TotalSeg	96.21 ± 1.68^0.046^ ^*^	88.30 ± 3.55^0.032^ ^*^	94.52 ± 2.61^0.009^ ^**^	96.45 ± 1.76^0.041^ ^*^	97.03 ± 1.82^0.018^ ^*^	97.52 ± 1.46^0.023^ ^*^
FS‐Mamba	**96.44 ± 1.57**	**88.31 ± 3.46**	**95.47 ± 2.40**	**96.71 ± 1.69**	95.92 ± 1.93	**97.77 ± 1.43**

Duodenum	Spinal Cord	Lungs	Heart	PTV	GTV	Mean
86.44 ± 4.22^0.001^ ^**^	95.10 ± 1.97^0.034^ ^*^	97.57 ± 1.63^0.007^ ^**^	87.52 ± 2.12^0.036^ ^*^	56.78 ± 7.09^0.001^ ^**^	78.21 ± 5.72^0.038^ ^*^	88.49 ± 3.58^0.002^ ^**^
88.59 ± 3.97^0.004^ ^**^	95.86 ± 1.81^0.040^ ^*^	98.10 ± 1.43^0.011^ ^*^	91.83 ± 1.77^0.006^ ^**^	75.92 ± 6.14^0.043^ ^*^	85.20 ± 5.17^0.013^ ^*^	91.89 ± 3.10^0.007^ ^**^
89.16 ± 3.87^0.004^ ^**^	96.46 ± 1.69^0.009^ ^**^	97.93 ± 1.49^0.015^ ^*^	90.53 ± 1.87^0.005^ ^**^	56.67 ± 7.18^0.001^ ^**^	75.13 ± 5.88^0.047^ ^*^	89.27 ± 3.51^0.004^ ^**^
86.94 ± 4.11^0.003^ ^**^	96.07 ± 1.75^0.007^ ^**^	97.95 ± 1.46^0.017^ ^*^	91.24 ± 1.82^0.005^ ^**^	72.76 ± 6.38^0.048^ ^*^	82.30 ± 5.34^0.004^ ^**^	91.24 ± 3.15^0.005^ ^**^
90.13 ± 3.75^0.005^ ^**^	96.42 ± 1.65^0.009^ ^**^	98.11 ± 1.42^0.042^ ^*^	92.17 ± 1.71^0.008^ ^**^	76.23 ± 6.08^0.036^ ^*^	85.25 ± 5.05^0.038^ ^*^	92.36 ± 2.88^0.021^ ^*^
**92.06 ± 3.49**	**96.69 ± 1.58**	98.07 ± 1.40	**92.65 ± 1.62**	**76.75 ± 6.03**	**85.29 ± 5.02**	**92.68 ± 2.79**

* *p* < 0.05

** *p* < 0.01

*** *p* < 0.001, versus FS‐Mamba (two‐tailed paired Wilcoxon signed‐rank test at the patient level). *P*‐value (DSC): Based on patient‐level average Dice across all organs.

**TABLE 4:** Quantitative comparison of segmentation models in terms of HD95 on the liver cancer dataset.

Model	HD95↓ (Liver Cancer Dataset)					
	Spleen	Pancreas	Stomach	Liver	Right Kidney	Left Kidney
UNet	11.47 ± 3.21^0.000^ ^***^	22.83 ± 6.27^0.000^ ^***^	14.62 ± 3.78^0.038^ ^*^	11.94 ± 2.89^0.009^ ^**^	10.32 ± 2.54^0.042^ ^*^	9.67 ± 2.63^0.027^ ^*^
nnUNet	9.21 ± 2.43^0.019^ ^*^	17.69 ± 4.64^0.006^ ^**^	11.27 ± 2.99^0.045^ ^*^	**7.95 ± 1.82** ^0.022^ ^*^	**7.13 ± 1.37** ^0.008^ ^**^	7.51 ± 1.73^0.033^ ^*^
TransUNet	10.29 ± 2.74^0.004^ ^**^	19.78 ± 5.31^0.041^ ^*^	12.53 ± 3.32^0.017^ ^*^	11.07 ± 2.61^0.035^ ^*^	8.76 ± 2.12^0.047^ ^*^	8.64 ± 2.18^0.005^ ^**^
Swin‐UMamba	10.55 ± 2.88^0.029^ ^*^	16.57 ± 4.19^0.031^ ^*^	10.52 ± 2.83^0.007^ ^**^	8.71 ± 2.09^0.044^ ^*^	8.25 ± 1.82^0.012^ ^*^	**6.87 ± 1.58** ^0.039^ ^*^
TotalSeg	9.96 ± 2.57^0.009^ ^*^	17.19 ± 4.42^0.025^ ^*^	6.90 ± 1.68^0.003^ ^**^	8.42 ± 1.95^0.015^ ^*^	**7.08 ± 1.29** ^0.048^ ^*^	7.19 ± 1.54^0.021^ ^*^
FS‐Mamba	**6.49 ± 1.56**	**12.81 ± 3.04**	**6.63 ± 1.52**	9.43 ± 2.33	8.94 ± 2.44	7.53 ± 1.95

Duodenum	Spinal Cord	Lungs	Heart	PTV	GTV	Mean
22.49 ± 6.31^0.011^ ^*^	12.67 ± 3.05^0.037^ ^*^	8.36 ± 2.27^0.002^ ^**^	17.68 ± 4.28^0.049^ ^*^	33.72 ± 8.62^0.023^ ^*^	25.68 ± 6.97^0.034^ ^*^	16.79 ± 2.43^0.009^ ^**^
18.82 ± 4.96^0.026^ ^*^	9.64 ± 2.49^0.043^ ^*^	**5.19 ± 1.18** ^0.014^ ^*^	13.87 ± 3.61^0.005^ ^**^	25.38 ± 6.44^0.030^ ^*^	22.46 ± 5.41^0.020^ ^*^	13.01 ± 1.78^0.040^ ^*^
20.34 ± 5.54^0.006^ ^**^	**7.91 ± 1.88** ^0.030^ ^*^	6.65 ± 1.99^0.045^ ^*^	13.60 ± 3.44^0.028^ ^*^	34.27 ± 8.29^0.004^ ^**^	27.82 ± 6.64^0.016^ ^*^	15.14 ± 2.11^0.036^ ^*^
17.69 ± 4.59^0.018^ ^*^	9.32 ± 2.27^0.007^ ^**^	5.91 ± 1.71^0.047^ ^*^	13.32 ± 3.19^0.039^ ^*^	26.79 ± 6.19^0.024^ ^*^	22.19 ± 5.19^0.008^ ^**^	13.06 ± 1.84^0.041^ ^*^
13.75 ± 3.34^0.003^ ^**^	9.88 ± 2.63^0.035^ ^*^	**5.12 ± 1.04** ^0.029^ ^*^	12.57 ± 2.96^0.044^ ^*^	23.07 ± 5.41^0.013^ ^*^	15.37 ± 3.32^0.031^ ^*^	11.38 ± 1.51^0.046^ ^*^
10.92 ± 2.37	9.03 ± 2.11	6.36 ± 1.93	**9.49 ± 2.12**	**18.67 ± 4.19**	15.72 ± 3.48	10.17 ± 1.29

* *p* < 0.05

** *p* < 0.01

*** *p* < 0.001, versus FS‐Mamba (two‐tailed paired Wilcoxon signed‐rank test at the patient level). *P*‐value (HD95): Based on patient‐level average HD95 distance across all organs.

To better align with clinical practice, 10 medical physicists performed a subjective evaluation of segmentation performance on the liver cancer dataset, including FS‐Mamba, UNet, nnUNet,^31^ TransUNet, Swin–UMamba, and the clinically widely used TotalSegmentator. The evaluation was based on 14 randomly selected cases (expanded from the original 4 shown in Figure 3) to improve statistical reliability. The assessment was conducted under blinded conditions: each contour set was labeled only with a random alphanumeric code (e.g., M001–M098), and the order of different contours within each case was randomized individually for each evaluator. The physicists reviewed complete 3D volumes rather than representative slices. In addition to model outputs, ground‐truth contours were also scored to provide a clinical reference baseline.

The scoring criteria (1–5) were defined based on boundary smoothness, anatomical integrity, and discrimination from adjacent organs: 5‐Perfect, no revisions needed, ready for direct use in planning; 4‐Nearly perfect, only minor flaws without impact on clinical use; 3‐Acceptable, but contains minor errors requiring revision; 2‐Numerous errors, extensive revision required for use; 1‐Completely unacceptable, must be redrawn. As presented in Table 5, the ground‐truth contours received a mean score of 4.94 ± 0.07, confirming high clinical acceptability of the reference standard. Among the models, UNet obtained the lowest average score (3.54 ± 0.12), with noticeable segmentation defects. Swin‐UMamba (3.81 ± 0.23) and nnUNet^31^ (4.01 ± 0.17) showed progressive improvements, while TransUNet achieved a score of 4.02 ± 0.14. TotalSegmentator, as a common clinical tool, yielded a stable average score of 4.39 ± 0.15, indicating good clinical utility. The proposed FS‐Mamba model achieved the highest average score among all methods (4.69 ± 0.12), closely approaching the ground‐truth level (4.94). These results suggest that FS‐Mamba's segmentation results align more closely with clinical requirements, demonstrating its potential value for liver tumor radiotherapy segmentation.

**TABLE 5:** Medical physicist model evaluation on liver cancer dataset. Overall subjective score: 5 Perfect, no revisions needed, ready for direct use in planning; 4 Nearly perfect, only minor flaws without impact on clinical use; 3 Acceptable, but contains minor errors requiring revision; 2 Numerous errors, extensive revision required for use; 1 Completely unacceptable, must be redrawn.

Model	Phy1	Phy2	Phy3	Phy4	Phy5	Phy6	Phy7	Phy8	Phy9	Phy10	Mean
Ground Truth	4.93 ± 0.27	5.00 ± 0.00	5.00 ± 0.00	4.93 ± 0.27	4.86 ± 0.36	4.93 ± 0.27	4.79 ± 0.58	5.00 ± 0.00	5.00 ± 0.00	5.00 ± 0.00	4.94 ± 0.07
Unet	3.71 ± 0.45	3.64 ± 0.48	3.50 ± 0.50	3.50 ± 0.50	3.50 ± 0.50	3.36 ± 0.48	3.43 ± 0.49	3.64 ± 0.48	3.43 ± 0.49	3.71 ± 0.45	3.54 ± 0.12
Swin‐UMamba	3.64 ± 0.61	3.64 ± 0.61	3.64 ± 0.61	3.86 ± 0.66	4.00 ± 0.71	4.21 ± 0.67	3.50 ± 0.50	3.64 ± 0.61	3.93 ± 0.66	4.00 ± 0.71	3.81 ± 0.23
nnUNet	4.07 ± 0.46	4.00 ± 0.53	4.21 ± 0.57	3.71 ± 0.45	4.21 ± 0.57	4.07 ± 0.59	3.86 ± 0.52	4.14 ± 0.52	3.86 ± 0.52	3.93 ± 0.59	4.01 ± 0.17
TransUNet	4.14 ± 0.52	3.93 ± 0.59	4.21 ± 0.67	4.00 ± 0.53	4.00 ± 0.53	4.07 ± 0.59	3.79 ± 0.57	4.21 ± 0.67	4.00 ± 0.71	3.86 ± 0.52	4.02 ± 0.14
TotalSeg	4.36 ± 0.61	4.29 ± 0.60	4.50 ± 0.50	4.29 ± 0.60	4.64 ± 0.48	4.29 ± 0.60	4.14 ± 0.64	4.36 ± 0.48	4.43 ± 0.49	4.57 ± 0.49	4.39 ± 0.15
FS‐Mamba	4.64 ± 0.48	4.79 ± 0.42	4.79 ± 0.42	4.93 ± 0.27	4.64 ± 0.48	4.50 ± 0.50	4.64 ± 0.48	4.57 ± 0.49	4.71 ± 0.45	4.71 ± 0.45	4.69 ± 0.12

For radiotherapy, particularly online adaptive workflows, computational efficiency is a core clinical requirement. As shown in Table 6, we compared the model parameters (Params), floating‐point operations (FLOPs), and average GPU inference time per case for all models under consistent hardware settings. UNet (40.89 M Params, 55.93 G FLOPs, 24.49 ms) and nnUNet^31^ (46.27 M Params, 60.18 G FLOPs, 29.72 ms) exhibited moderate computational overhead. TransUNet had a high computational cost with 86.35 M parameters, 153.29 G FLOPs, and an inference time of 76.71 ms. Swin–UMamba (27.39 M parameters, 48.90 G FLOPs, 24.75 ms) achieved a relatively lightweight design. TotalSegmentator, a widely used clinical tool, exhibited the highest computational burden among all methods (92.50 M parameters, 410.16 GFLOPs, 131.28 ms), which may limit its applicability in time‐sensitive online adaptive scenarios. Our proposed FS‐Mamba achieved the lowest computational cost across all metrics, with only 20.57 M parameters, 31.94 GFLOPs, and the shortest inference time of 20.16 ms. While maintaining leading segmentation accuracy, it strikes a favorable balance between precision and efficiency, which can effectively meet the strict time constraints of clinical online adaptive radiotherapy.

**TABLE 6:** Computational comparison on the liver cancer dataset.

Model	Num of params (M)	FLOPs(G)	GPU T.(ms)
UNet	40.89	55.93	24.49
nnUNet	46.27	60.18	29.72
TotalSeg	92.50	410.16	131.28
TransUNet	86.35	153.29	76.71
Swin‐UMamba	27.39	48.90	24.75
FS‐Mamba	20.57	31.94	20.16

### 3.4 Ablation study

To verify the independent contributions and synergistic benefits of the three core innovative components of FS‐Mamba, namely the FS‐Scanning, frequency‐domain modeling branch, and long‐memory SSM (LSSM), we conducted univariate ablation experiments on the Synapse abdominal multi‐organ segmentation dataset. Four ablation variants were set up: WithoutFFT (with the frequency‐domain branch removed), WithoutA’(with the A’ matrix augmentation removed, i.e., replacing the LSSM with the SSM), WithoutFscan (with FS‐Scanning replaced by the original Cross‐Scanning), and WithoutLF (with both the long‐memory SSM and FS‐Scanning replaced by SSM and Cross‐Scanning simultaneously).

**FIGURE 4:**
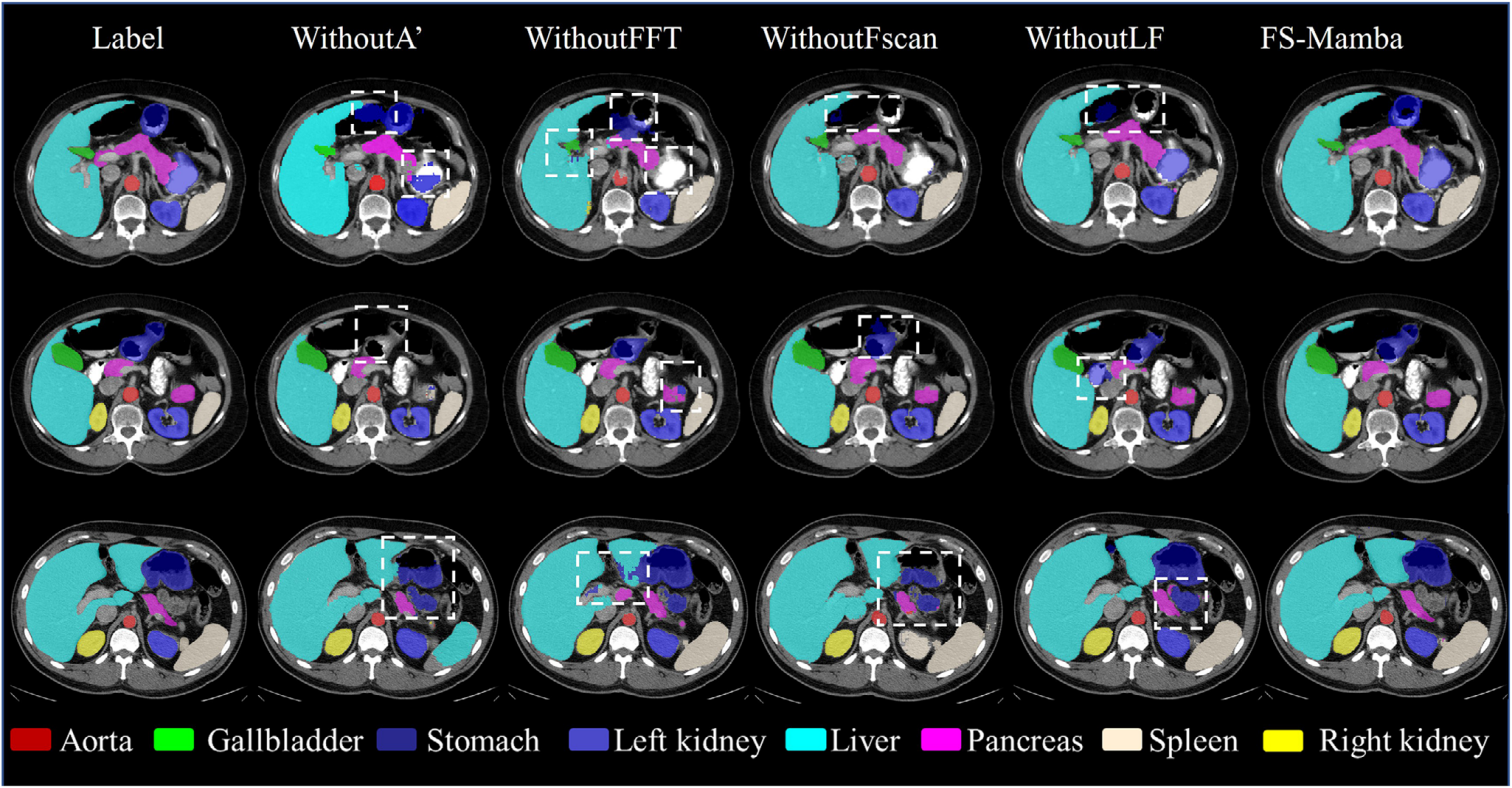
Qualitative comparison of segmentation results under different ablation settings on the Synapse dataset.

A systematic analysis was performed combining the qualitative visualization results in Figure 4 and the quantitative metrics in Tables 7 and 8. The segmentation results of the full FS‐Mamba are highly consistent with the ground truth, achieving accurate segmentation for high‐contrast organs such as the liver and aorta, as well as low‐contrast fine structures including the gallbladder and pancreas. In contrast, the ablation variants all exhibited varying degrees of structural omission, boundary blurring, and mis‐segmentation, among which the variant with the A’ matrix removed showed the most severe performance degradation. The quantitative results in Tables 7 and 8 further confirm that the full FS‐Mamba achieves the optimal performance (Dice of 81.28%, HD95 of 14.32, and 20.57 M parameters). Specifically, removing the A’ matrix (WithoutA’, 16.53 M parameters) leads to a sharp drop of 12.25 percentage points in Dice (to 69.03%) and a large increase in HD95 (to 30.66). Removing the frequency‐domain branch (WithoutFFT, 18.29 M parameters) results in a 6.10 percentage point decrease in Dice (to 75.18%) and an HD95 of 24.43. Notably, removing the LF module (WithoutLF, 26.59 M parameters) causes a 2.38 percentage point reduction in Dice (to 78.90%) and an HD95 increase to 19.82, while removing the FS‐Scanning module (WithoutFscan, 38.06 M parameters) yields a slightly smaller performance drop with a Dice of 79.23% and HD95 of 17.41, but incurs a much higher computational cost. The above results fully validate the necessity and synergistic advantages of the three core modules, which enable the simultaneous optimization of segmentation accuracy and computational efficiency.

**TABLE 7:** Quantitative comparison of ablation models in terms of Dice on the Synapse dataset.

Model	Dice↑ (Synapse Dataset)					
	Aorta	Gallbladder	Kidney (L)	Kidney (R)	Liver	Pancreas
WithoutA'	80.34 ± 4.21^0.000^ ^***^	58.92 ± 5.07^0.000^ ^***^	71.06 ± 3.88^0.000^ ^***^	72.55 ± 4.63^0.000^ ^***^	84.21 ± 2.05^0.000^ ^***^	45.78 ± 6.12^0.000^ ^***^
WithoutFFT	86.57 ± 2.33^0.002^ ^**^	61.44 ± 4.89^0.001^ ^**^	80.33 ± 2.95^0.003^ ^**^	70.12 ± 5.41^0.000^ ^***^	88.09 ± 1.77^0.004^ ^**^	53.91 ± 4.30^0.001^ ^**^
WithoutFscan	83.22 ± 3.07^0.018^ ^*^	67.85 ± 3.90^0.029^ ^*^	79.46 ± 4.02^0.013^ ^*^	75.33 ± 2.98^0.022^ ^*^	90.77 ± 1.54^0.035^ ^*^	55.68 ± 4.45^0.019^ ^*^
WithoutLF	88.91 ± 1.72^0.041^ ^*^	64.27 ± 4.05^0.011^ ^*^	82.14 ± 2.68^0.026^ ^*^	78.86 ± 3.17^0.033^ ^*^	91.43 ± 1.39^0.038^ ^*^	53.24 ± 5.08^0.014^ ^*^
FS‐Mamba	**90.02 ± 1.63**	**70.15 ± 3.47**	**84.42 ± 2.57**	**80.67 ± 2.89**	**94.24 ± 0.98**	**60.27 ± 4.05**

Spleen	Stomach	Mean	Params(M)			
76.43 ± 4.95^0.000^ ^***^	70.22 ± 5.08^0.000^ ^***^	69.03 ± 3.77^0.000^ ^***^	16.53			
80.17 ± 3.31^0.002^ ^**^	73.85 ± 4.26^0.001^ ^**^	75.18 ± 2.94^0.002^ ^**^	18.29			
83.59 ± 2.24^0.025^ ^*^	77.94 ± 3.50^0.031^ ^*^	79.23 ± 2.80^0.022^ ^*^	38.06			
85.12 ± 2.17^0.035^ ^*^	79.46 ± 3.37^0.027^ ^*^	78.90 ± 2.59^0.029^ ^*^	26.59			
**87.45 ± 1.78**	**83.01 ± 3.04**	**81.28 ± 2.65**	**20.57**			

* *p* < 0.05

** *p* < 0.01

*** *p* < 0.001, versus FS‐Mamba (two‐tailed paired Wilcoxon signed‐rank test at the patient level). *P*‐value (DSC): Based on patient‐level average Dice across all organs.

**TABLE 8:** Quantitative comparison of ablation models in terms of HD95 on the Synapse dataset.

Model	HD95↓ (Synapse Dataset)					
	Aorta	Gallbladder	Kidney (L)	Kidney (R)	Liver	Pancreas
WithoutA'	25.81 ± 6.35^0.000^ ^***^	42.27 ± 8.82^0.000^ ^***^	31.52 ± 5.97^0.000^ ^***^	28.97 ± 7.11^0.000^ ^***^	18.42 ± 3.49^0.000^ ^***^	48.86 ± 9.56^0.000^ ^***^
WithoutFFT	19.92 ± 4.12^0.^003^**^	35.76 ± 7.19^0.001^ ^**^	24.65 ± 4.27^0.002^ ^**^	30.38 ± 7.62^0.001^ ^**^	15.01 ± 2.92^0.004^ ^**^	31.63 ± 7.28^0.001^ ^**^
WithoutFscan	22.57 ± 5.24^0.038^ ^*^	24.99 ± 5.21^0.026^ ^*^	27.51 ± 5.34^0.032^ ^*^	22.29 ± 5.42^0.041^ ^*^	13.96 ± 2.71^0.035^ ^*^	26.97 ± 6.48^0.022^ ^*^
WithoutLF	16.44 ± 3.70^0.033^ ^*^	28.41 ± 6.58^0.017^ ^*^	19.96 ± 3.80^0.021^ ^*^	17.76 ± 4.17^0.029^ ^*^	12.12 ± 2.15^0.033^ ^*^	34.39 ± 7.94^0.018^ ^*^
FS‐Mamba	**12.24 ± 1.67**	**17.81 ± 3.89**	**11.63 ± 2.15**	**14.43 ± 2.68**	**8.94 ± 3.12**	**17.92 ± 4.23**
Spleen	Stomach	Mean				
22.64 ± 5.48^0.000^ ^***^	26.87 ± 6.17^0.000^ ^***^	30.66 ± 4.93^0.000^ ^***^				
20.52 ± 4.91^0.002^ ^**^	20.52 ± 4.91^0.001^ ^**^	24.43 ± 3.80^0.002^ ^**^				
17.98 ± 4.09^0.019^ ^*^	17.31 ± 3.99^0.027^ ^*^	17.41 ± 2.48^0.031^ ^*^				
14.84 ± 3.26^0.033^ ^*^	15.76 ± 3.12^0.038^ ^*^	19.82 ± 3.02^0.022^ ^*^				
**12.53 ± 2.04**	**14.03 ± 2.56**	**14.32 ± 2.40**				

* *p* < 0.05

** *p* < 0.01

*** *p* < 0.001, versus FS‐Mamba (two‐tailed paired Wilcoxon signed‐rank test at the patient level). *P*‐value (HD95): Based on patient‐level average HD95 distance across all organs.

**FIGURE 5:**
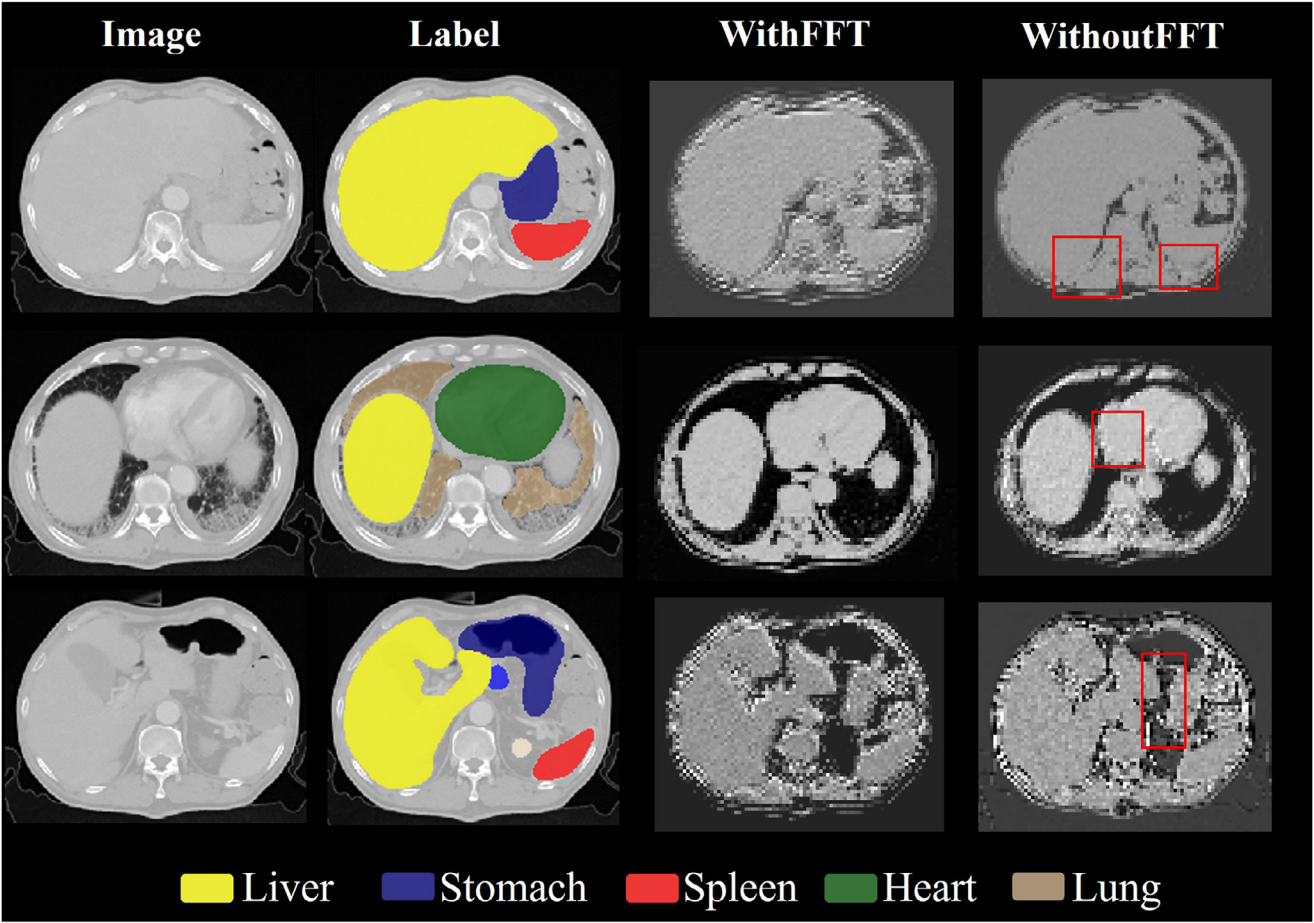
Comparison of 128 × 128‐resolution feature maps with and without the frequency domain branch on the liver cancer dataset. Feature maps show clearer boundary division after frequency domain branch‐assisted feature extraction.

To provide a more intuitive demonstration of the frequency domain branch's role, we present visualizations of 128 × 128‐resolution feature maps within the encoder^21^ with and without this branch on the liver cancer dataset. As shown in Figure 5, the inclusion of the frequency domain branch enables the FSM Block to distinguish boundaries more distinctly, thereby enhancing segmentation performance through clearer feature representation.

## 4 DISCUSSION

To address the long‐range modeling limitations of CNNs and the high computational complexity of Transformers, we designed FS‐Mamba, a Mamba‐based medical image segmentation model. FS‐Mamba introduces the Frequency‐Long SSM Block, which for the first time combines frequency‐domain features with time‐domain features extracted by Mamba and applies them to the medical image segmentation task. Additionally, to ensure that Mamba retains more details from front‐end features in long sequences, we developed the Long‐Memory SSM algorithm, which enhances the model's memorization capability through the A matrix. Moreover, when faced with various scanning methods, rather than adding additional scans, we explored how to reduce the number of scans without compromising results. Based on the theory that SSMs generalize more strongly to earlier features and lose more details over time, we designed the FS‐Scanning to prioritize more important features in the back‐end and minimize detail loss due to generalization. This approach uses only one row and one column scan, achieving the same or better results than the original four cross‐scans when combined with Long‐Memory SSM and frequency‐domain assistance. We validated the performance of the model on the CT‐ORG, Synapse, and liver cancer datasets, and experimentally demonstrated that FS‐Mamba has unique advantages in both multi‐organ segmentation and fundus vascular segmentation tasks.

In addition to achieving statistically significant improvements in segmentation metrics, we further elaborated on the clinical application of FS‐Mamba in the radiation therapy of liver cancer. In modern adaptive radiotherapy (ART), commercial adaptive linear accelerators such as the Elekta Unity and Varian Ethos primarily rely on deformable image registration (DIR) technology to map reference contours onto daily treatment images, which must then be manually corrected by radiation oncologists.^34^, ^35^ Although DIR‐based contour propagation eliminates the need for fully manual repositioning, its ability to alleviate clinicians’ workload is often limited due to the substantial review and editing required.^36^ In contrast, the proposed FS‐Mamba model can perform fully automated de novo segmentation of all abdominal organs in a short time, and its performance on all evaluated organs is statistically equivalent to expert manual contouring (expert score 4.69/5). The significant reduction in contouring time has greatly shortened the overall treatment cycle, opening up new possibilities for efficient online adaptive radiotherapy.

The current application of the FS‐Mamba model faces two limitations: in terms of the learning paradigm, its core architecture relies on supervised learning and requires segmentation masks with pixel‐level precision. However, medical image annotation is highly specialized and costly, and must be validated with a Dice coefficient of 0.92. For the segmentation of rare diseases or complex anatomical structures, studies have shown that insufficient annotated training samples can lead to reduced accuracy in tumor boundary localization, weakened generalization capabilities, and increased clinical risks.^4^, ^37^ Furthermore, clinical data collection is constrained by ethics review cycles, patient recruitment rates, and device compatibility, further exacerbating the imbalance between data supply and demand. Regarding modality adaptability, while the model performs excellently on CT image tasks, its effectiveness on MRI or other imaging modalities has not yet been validated, limiting its expansion into a comprehensive medical image analysis platform.

In the future, we plan to explore further how to reduce the number of scans in Mamba without compromising results, potentially by combining it with other methods to compensate. We will also investigate new approaches to integrating frequency domain features with Mamba to discover more advantageous combinations. Furthermore, we aim to extend the model to more segmentation tasks, such as skin lesion segmentation and brain segmentation, to fully validate its performance.

## 5 CONCLUSION

In this work, we propose FS‐Mamba, a lightweight, high‐precision U‐shaped network dedicated to CT image segmentation for liver cancer radiotherapy. Its core innovation lies in the synergistic design of a frequency‐domain feature fusion module and a long‐memory state space model, which effectively tackles the common challenges of blurred boundaries in low‐contrast organs and difficult fine‐structure segmentation in abdominal CT images. Experiments on our in‐house liver cancer dataset show that FS‐Mamba achieves leading segmentation performance, with an average Dice coefficient of 92.68% across 12 anatomical structures, ranking first in 10 out of 12 segmentation tasks. Meanwhile, the model maintains an extremely lightweight design with only 20.57 M parameters and 31.94 G FLOPs, and delivers the shortest per‐case inference time of 20.16 ms, outperforming all comparative models in computational efficiency. Both quantitative metrics and subjective clinical evaluation (with the highest mean score of 4.69/5) confirm that FS‐Mamba fully meets the dual clinical requirements of segmentation accuracy and efficiency, especially for the strict time constraints of online adaptive radiotherapy workflows. This model provides a reliable technical solution for automated organ and tumor target contouring, and has promising application potential for advancing precision liver cancer radiotherapy.

## AUTHOR CONTRIBUTIONS

Peijun Yin and Xueren Zhang conceived and designed the FS‐Mamba model, with Peijun Yin focusing on the FS‐Scanning and Xueren Zhang on the long‐memory SSM mechanism. Peijun Yin, Xueren Zhang, and Qingtao Qiu drafted the initial manuscript and made substantial revisions. Qingtao Qiu participated in the design of experimental protocols, assisted in analyzing the segmentation results of multiple datasets, and contributed to the optimization of the model's performance evaluation metrics. Xin Liu collected and curated the liver cancer dataset, including the labeling of OARs and target volumes. Zekun Jiang developed the software framework for model training, inference, and data preprocessing, and optimized computational efficiency. Yong Yin supervised the research direction, refined the model design based on clinical needs, and revised the manuscript. Zhenjiang Li led the project, conceptualized the integration of frequency domain features with state space models, oversaw experimental design and validation, and finalized the manuscript. All authors read and approved the final manuscript.

## CONFLICT OF INTEREST STATEMENT

The authors declare no conflicts of interest.

## References

[1] Ma J , Wang B . Towards foundation models of biological image segmentation. Nat Methods. 2023;20(7):953‐955. doi:10.1038/s41592‐023‐01885‐0 37433999 10.1038/s41592-023-01885-0

[2] Minaee S , Boykov Y , Porikli F , Plaza A , Kehtarnavaz N , Terzopoulos D . Image segmentation using deep learning: a survey. IEEE Trans Pattern Anal Mach Intell. 2021;44(7):3523‐3542. 10.1109/TPAMI.2021.3059968 33596172

[3] Sharp G , Fritscher KD , Pekar V , et al. Vision 20/20: perspectives on automated image segmentation for radiotherapy. Med phys. 2014;41(5):050902. doi:10.1118/1.4871620 24784366 10.1118/1.4871620 PMC4000389

[4] Litjens G , Kooi T , Bejnordi BE , et al. A survey on deep learning in medical image analysis. Med image anal. 2017;42:60‐88. doi:10.1016/j.media.2017.07.005 28778026 10.1016/j.media.2017.07.005

[5] Heron DE , Ferris RL , Karamouzis M , et al. Stereotactic body radiotherapy for recurrent squamous cell carcinoma of the head and neck: results of a phase I dose‐escalation trial. Int J Radiat Oncol Biol Phys. 2009;75(5):1493‐1500. doi:10.1016/j.ijrobp.2008.12.075 19464819 10.1016/j.ijrobp.2008.12.075

[6] Cengiz M , Özyiğit G , Yazici G , et al. Salvage reirradiaton with stereotactic body radiotherapy for locally recurrent head‐and‐neck tumors. Int J Radiat Oncol Biol Phys. 2011;81(1):104‐109. doi:10.1016/j.ijrobp.2010.04.027 20675075 10.1016/j.ijrobp.2010.04.027

[7] Carsuzaa F , Lapeyre M , Gregoire V , et al. Recommendations for postoperative radiotherapy in head & neck squamous cell carcinoma in the presence of flaps: a gortec internationally‐reviewed hncig‐endorsed consensus. Radiother Oncol. 2021;160:140‐147. doi:10.1016/j.radonc.2021.04.026 33984351 10.1016/j.radonc.2021.04.026

[8] Witt JS , Rosenberg SA , Bassetti MF . MRI‐guided adaptive radiotherapy for liver tumours: visualising the future. Lancet Oncol. 2020;21(2):74‐82. doi:10.1016/S1470‐2045(20)30034‐6 10.1016/S1470-2045(20)30034-6 32007208

[9] Shi F , Hu W , Wu J , et al. Deep learning empowered volume delineation of whole‐body organs‐at‐risk for accelerated radiotherapy. Nat Commun. 2022;13(1):6566. doi:10.1038/s41467‐022‐34257‐x 36323677 10.1038/s41467-022-34257-x PMC9630370

[10] Marschner S , Datar M , Gaasch A , et al. A deep image‐to‐image network organ segmentation algorithm for radiation treatment planning: principles and evaluation. Radiat Oncol. 2022;17(1):129. doi:10.1186/s13014‐022‐02102‐6 35869525 10.1186/s13014-022-02102-6 PMC9308364

[11] Navarro F , Sasahara G , Shit S , et al. A unified 3d framework for organs‐at‐risk localization and segmentation for radiation therapy planning. In: 2022 44th Annual International Conference of the IEEE Engineering in Medicine & Biology Society (EMBC) , IEEE; 2022:1544‐1547. doi:10.1109/EMBC48229.2022.9871680 10.1109/EMBC48229.2022.9871680 36086554

[12] Zhao H , Meng B , Dohopolski M , et al. Segmentation of targets and organs at risk for CBCT based online adaptive radiotherapy using recurrent neural networks: a clinical evaluation. Int J Radiat Oncol Biol Phys. 2022;114(3):558‐559. doi:10.1016/j.ijrobp.2022.07.2197

[13] Ding J , Zhang Y , Amjad A , Xu J , Thill D , Li XA . Automatic contour refinement for deep learning auto‐segmentation of complex organs in MRI‐guided adaptive radiation therapy. Adv Radiat Oncol. 2022;7(5):100968. doi:10.1016/j.adro.2022.100968 35847549 10.1016/j.adro.2022.100968 PMC9280040

[14] Webster M , Podgorsak A , Li F , et al. New approaches in radiotherapy. Cancers. 2025;17(12):1980. doi:10.3390/cancers17121980 40563630 10.3390/cancers17121980 PMC12190917

[15] Rammohan N , Randall JW , Yadav P . History of technological advancements towards MR‐Linac: the future of image‐guided radiotherapy. J Clin Med. 2022;11(16):4730. doi:10.3390/jcm11164730 36012969 10.3390/jcm11164730 PMC9409689

[16] Xiao F , Cai J , Zhou X , Zhou L , Song T , Li Y . Transdose: a transformerbased UNet model for fast and accurate dose calculation for MR‐Linacs. Phys Med Biol. 2022;67(12):125013. doi:10.1088/1361‐6560/ac7376 10.1088/1361-6560/ac7376 35613559

[17] Ronneberger O , Fischer P , Brox T . UNet: convolutional networks for biomedical image segmentation. In: Medical Image Computing and Computer‐assisted intervention–MICCAI 2015: 18th International Conference , Munich, Germany, Proceedings, Part III 18, Springer; 2015:234‐241.

[18] Vaswani A , Shazeer N , Parmar N , et al. Attention is all you need. Adv Neural Inf Process Syst. 2017;30.

[19] Devlin J , Chang M‐W , Lee K , Toutanova K . BERT: Pre‐training of deep bidirectional transformers for language understanding. ACL Anthol. 2018. preprint arXiv:1810.04805.

[20] Dosovitskiy A , Beyer L , Kolesnikov A , et al. An image is worth 16 × 16 words: transformers for image recognition at scale. arXiv. 2020. preprint arXiv:2010.11929.

[21] Chen J , Lu Y , Yu Q , et al. Transunet: transformers make strong encoders for medical image segmentation. arXiv. 2021. preprint arXiv:2102.04306.

[22] Cao H , Wang Y , Chen J , et al. Swinunet: UNet‐like pure transformer for medical image segmentation. In: European Conference on Computer Vision , Springer; 2022:205‐218.

[23] Wang Z , Su M , Zheng J‐Q , Liu Y . Densely connected swin‐UNet for multiscale information aggregation in medical image segmentation. In: 2023 IEEE International Conference on Image Processing (ICIP) , IEEE; 2023:940‐944. doi:10.1109/ICIP49359.2023.10222451

[24] Xing Z , Ye T , Yang Y , Liu G , Zhu L . Segmamba: long‐range sequential modeling mamba for 3d medical image segmentation. In: International Conference on Medical Image Computing and Computer‐Assisted Intervention , Springer; 2024:578‐588.

[25] Ma J , Li F , Wang B . U‐Mamba: enhancing long‐range dependency for biomedical image segmentation. arXiv. 2024. preprint arXiv:2401.04722.

[26] Gu A , Dao T . Mamba: linear‐time sequence modeling with selective state spaces. arXiv. 2023. preprint arXiv:2312.00752.

[27] Zhu L , Liao B , Zhang Q , Wang X , Liu W , Wang X . Vision mamba: efficient visual representation learning with bidirectional state space model. arXiv. 2024. preprint arXiv:2401.09417.

[28] Xu R , Yang S , Wang Y , Du B , Chen H . A survey on vision mamba: models, applications and challenges. arXiv 2024.

[29] Rister B , Yi D , Shivakumar K , Nobashi T , Rubin DL . CT‐org, a new dataset for multiple organ segmentation in computed tomography. Sci Data. 2020;7(1):381. doi:10.1038/s41597‐020‐00715‐8 33177518 10.1038/s41597-020-00715-8 PMC7658204

[30] Landman BA , Huang Z , Geva A , Wang A , Reeves L , Dawant B , et al. Multi‐atlas segmentation of the whole abdomen from CT. In: 2015 IEEE 12th International Symposium on Biomedical Imaging (ISBI) , IEEE; 2015:100‐103.

[31] Isensee F , Jaeger PF , Kohl SA , Petersen J , Maier‐Hein KH . nnUNet: a self‐configuring method for deep learning‐based biomedical image segmentation. Nat Methods. 2021;18(2):203‐211. doi:10.1038/s41592‐020‐01008‐z 33288961 10.1038/s41592-020-01008-z

[32] Liu J , Yang H , Zhou H‐Y , et al. Swin‐UMamba: Mamba‐based Unet with ImageNet‐based pretraining. arXiv. 2024. preprint arXiv:2402.03302.

[33] Wasserthal J , Meyer M , Breit HC , Cyriac J , Yang S , Segeroth M . TotalSegmentator: robust segmentation of 104 anatomical structures in CT images. Med Image Anal. 2023;83:102699. 10.1148/ryai.230024 PMC10546353 37795137

[34] Winkel D , Bol GH , Kroon PS , et al. Adaptive radiotherapy: the elekta unity MR‐Linac concept. Clin Transl Radiat Oncol. 2019;18:54‐59. doi:10.1016/j.ctro.2019.04.001 31341976 10.1016/j.ctro.2019.04.001 PMC6630157

[35] Byrne M , Archibald‐Heeren B , Hu Y , et al. Varian ethos online adaptive radio‐therapy for prostate cancer: early results of contouring accuracy, treatment plan quality, and treatment time. J Appl Clin Med Phys. 2022;23(1):13479. doi:10.1002/acm2.13479 10.1002/acm2.13479 PMC8803282 34846098

[36] Green OL , Henke LE , Hugo GD . Practical clinical workflows for online and offline adaptive radiation therapy. Semin Radiat Oncol. 2019;29:219‐227. doi:10.1016/j.semradonc.2019.02.004 31027639 10.1016/j.semradonc.2019.02.004 PMC6487881

[37] Rieke N , Hancox J , Li W , et al. The future of digital health with federated learning. NPJ Digital Med. 2020;3(1):119. doi:10.1038/s41746‐020‐00323‐1 10.1038/s41746-020-00323-1 PMC7490367 33015372

